# Clinical Profile of Neonates with Respiratory Distress in a Tertiary Care Hospital

**DOI:** 10.31729/jnma.4770

**Published:** 2019-12-31

**Authors:** Anita Lamichhane, Kiran Panthee, Sharmila Gurung

**Affiliations:** 1 Department of Pediatrics, Lumbini Medical College, Pravas, Palpa, Nepal; 2 Department of Pediatrics, Devdaha Medical College, Bhaluhi, Rupandehi, Nepal; 3 Department of Forensic Medicine, Devdaha Medical College, Bhaluhi, Rupandehi, Nepal

**Keywords:** *neonatal intensive care unit*, *neonates*, *respiratory distress*

## Abstract

**Introduction:**

Respiratory distress in newborns is a very common reason for admission in Neonatal Intensive Care Unit which may be transient or pathological; morbidity is high if not prompted for early diagnosis and treatment. The present study is undertaken to find out the clinical profile of neonates with respiratory distress in infants in a tertiary care hospital in western Nepal.

**Methods:**

A descriptive cross-sectional study was carried out in a tertiary care hospital in the western region of Nepal from April 2017 to March 2018 after approval from the Institutional Review Committee. Convenience sampling was done. Data were collected from the study population after taking consent and entered in a predesigned proforma. Data was then entered in a Statistical Package for Social Sciences for further calculations.

**Results:**

Tachypnea was the most common presentation 77 (69.36%). Out of 1694 live deliveries during the study period, the prevalence of respiratory distress was 6.55% in the total live deliveries while 30.83% in admitted cases in Neonatal Intensive Care Unit. Survival rate was 95.50% while mortality rate accounted for 4.50%.

**Conclusions:**

Perinatal asphyxia accounted for the commonest cause of respiratory distress. To lessen the morbidity and mortality of the neonates with respiratory distress it is advocated that we practice proper and timely neonatal resuscitation, recognize the risk factors as early as possible so that perinatal asphyxia can be minimized.

## INTRODUCTION

Globally respiratory distress in newborns is the most common presentation which requires hospital admission. Saeed Z et al. described respiratory distress as the most common presenting problem encountered within the first 48-72 hours of life with a prevalence of 4.24% in neonates.^1^

The most common causes of respiratory distress include Transient Tachypnea of the Newborn (TTN), Hyaline Membrane Disease (HMD), Birth asphyxia, Pneumonia and Meconium Aspiration Syndrome (MAS).^2^ There are studies which have reported the incidence of respiratory distress in newborn babies to be ranging from 3.9 to 8 % in admitted patients in Neonatal Intensive Care Unit (NICU).^3,4^

There has been tremendous advances in the treatment of neonatal respiratory distress syndrome but very few clinical studies have been conducted in our country. So this study was undertaken to find out the clinical profile of babies with respiratory distress.

## METHODS

This descriptive cross-sectional study was conducted in the neonatal intensive care unit (NICU) of Devdaha Medical College and Teaching Hospital (LMCTH) in Western Nepal from April 2017 to March 2018 at Devdaha Medical College, Bhaluhi, Rupandehi, Nepal after taking approval from the institutional review committee (IRC) of the college.

The present study included all the term and preterm neonates who developed respiratory distress within 48 hours after birth, both inborn and outborn and singleton babies. Those neonates who developed respiratory distress 48 hours after birth and having congenital anomalies like congenital diaphragmatic hernia, anencephaly, and meningomyelocele and twin babies were excluded from the study. A written consent was obtained from the mother for the participation in the study for their newborn neonates.

Respiratory distress was diagnosed based clinically when two out of the following were present.

- Tachypnea (respiratory rate>60 breaths/min)
- Nasal flaring
- Intercostal recession
- Subcostal recession
- Grunting

A detailed history regarding birth weight, maternal age, gestational age at the time of delivery, sex, mode and place of delivery, maternal risk factors, meconium stained liquor, was taken from the mother and entered in the predesigned proforma. After admission in the NICU, babies were thoroughly examined and monitored daily till discharge from the hospital or death. They were treated as per the NICU protocols of the hospital having specified indications for oxygen therapy, CPAP, mechanical ventilation. Relevant investigations like full blood count, C-reactive protein, blood glucose, blood cultures, blood gases, chest x-ray were sent. In case of death, the cause of mortality was recorded. Severity of distress was assessed by Anderson Silverman Score.^5^

Based on this score, the babies with respiratory distress were categorized in three groups. These were mild (1-3 score), moderate (4-6 score), severe (>6 score).

Data was checked for any errors or inconsistencies, then entered in Microsoft Excel sheets and analysed using Statistical Package for Social Sciences (SPSS) version 25.0.

Convenience sampling and the sample size was calculated using the formula,^6^

$$\begin{array}{ll} \text{n} & ={\text{Z}}^{\text{2}}\times \text{(p}\times \text{q)}/{\text{e}}^{\text{2}}\\ & =\text{1}{\text{.96}}^{\text{2}}\times \text{0.08}\times \text{(1}-\text{0.08)}/\text{0}{\text{.05}}^{\text{2}}\\ & =113 \end{array}$$

where,

n= required sample size
p= prevalence of respiratory distress (8%)^3^
q= 1-p
e= margin of error, 5%
Z= 1.96 at 95 % CI

Total sample size was calculated to be 113.

## RESULTS

The most common presentation of respiratory distress was tachypnea, 77 (69.36%) (Table 1). Out of 1694 live deliveries during the study period, 360 (21.25%) neonates were admitted to NICU. Out of them 168 (46.6%) babies fulfilling the inclusion and exclusion criteria were included in the study. Mothers of fifty seven babies did not give consent for the study. So, 111 (30.83%) cases fulfilling the inclusion and exclusion criteria were enrolled in the study giving prevalance of 6.55% in the total live deliveries. The mean weight of the babies was 2.25±0.63 kg. Respiratory distress was common (61.26%) in the low birth weight babies (<2.5Kg). The demographic profile of the babies are shown (Table 2). Anderson Silverman scoring showed that there were 24 (21.62%) neonates in mild category, 75 (67.57%) in moderate category while severe category had 12 (10.82%) neonates. Survival rate was 106 (95.50%) while mortality accounted for 05 (4.50%) (Table 3).

**Table 1:** Showing the clinical features of neonates with respiratory distress.

Clinical presentation	n (%)
Tachypnea	77 (69.36)
Chest in drawing	10 (09.01)
Cyanosis	3 (2.70)
Poor perfusion	3 (2.70)
Grunting	14 (12.61)
Nasal flaring	4 (3.60)

**Table 2:** Showing the demographic profile respiratory distress of the study population.

Characteristics	n (% )
Outcome	
Live	106 (95.50)
Dead	5(4.50)
Birth weight	
1-1.4 kg	12 (10.81)
1.5-2.4 kg	56 (50.45)
2.5-3.4 kg	43 (38.74)
Mode of delivery	
Normal delivery	51 (49.95)
Caesarean	57 (51.35)
Vacuum	3 (2.70)
Sex	
Male	67 (60.36)
Female	43 (38.74)
Ambiguous genitalia	1 (0.90)
Gestational age	
Term (≥37 weeks)	45 (40.54)
Preterm (<37 weeks)	66 (59.46)

**Table 3:** Showing the outcome of the neonates with severity of respiratory distress.

	n (%)	Survived	Expired
Mild respiratory distress (1-3)	24 (21.62)	23 (99.83)	1 (4.17)
Moderate respiratory distress (4-6)	75 (67.57)	73 (97.33%)	2 (2.67)
Severe respiratory distress (9>6)	12 (10.81)	9 (8.11)	2 (1.80)
Total	111 (100 %)	106 (95.50%)	5 (4.50%)

## DISCUSSION

During the study period out of 1694 deliveries and 360 NICU admissions, 111 (30.83%) neonates developed respiratory distress making an incidence of 6.55% in total population and 30.83% in admitted babies. This is similar to studies done in Nepal which showed the incidence ranging from 3.9 % to 8.0% in total population.^3,5^ There are some other studies confirming respiratory distress is common in neonates and occurs in approximately 7% of babies during the neonatal period.^7,8^ Another study done by Malik et al. showed 47.2% incidence of respiratory distress of NICU admissions.^9^

Other studies had different incidences ranging from 20 to 50%.^10,11^ This is quite similar to studies done in India (60.62%)^12^ and Bangladesh (505.3%).^13^ This high rate may be due to the vulnerability of the LBW babies predisposing to respiratory problems and infections. Tachypnea was the most common presentation (69.36%). Most of the babies with respiratory distress were delivered by caesarean section (51.35%) while normal vaginal delivery accounted for 49.95%. This is in contrast to a study done in India^11^ and Nepal^7^ which showed that vaginal delivery was more commonly associated with the development of respiratory distress in newborn babies. This high rate of caesarean section associated with respiratory distress may be due to that fluid clearance may be a bit delayed in babies contributing to developing distress.

There was male preponderance in our study (60.36%) as compared to female babies (38.74%).^14^ This is comparable to another study done by Miller et al. which showed incidence of respiratory distress was almost three times higher in male babies than in female babies. Similar studies by Kanodia,^15^ Mmbaga^16^ and Shah GS^2^ reported male predominance. Male babies are likely to be affected 2-4 times more than female babies.^17^ There is a presence of gender bias still in all regions and cultures and hence male babies are brought to the hospital in more number. This may be one of the reason for male preponderance.

The survival rate was good in our study (95.50%) while mortality accounted for 4.50%. This low mortality may be due to prompt recognition of the symptoms and treatment.

There were certain limitations of our study. It was a small sample sized hospital based study done in a certain limited time. A large population based study will be needed to strengthen our study.

## CONCLUSIONS

Respiratory distress embraced 6.55% of all total live deliveries while it constituted 30.83% of all NICU admission. To lessen the morbidity and mortality of the neonates with respiratory distress it is advocated that we practice proper and timely neonatal resuscitation, recognize the risk factors as early as possible so that perinatal asphyxia can be minimized. We should also advocate good obstetric care.

## Conflict of Interest

**None.**
